# Shifting Evaluation Windows: Predictable Forward Primes with Long SOAs Eliminate the Impact of Backward Primes

**DOI:** 10.1371/journal.pone.0054739

**Published:** 2013-01-24

**Authors:** Daniel A. Fockenberg, Sander L. Koole, Daniël Lakens, Gün R. Semin

**Affiliations:** 1 Institut für Psychologie, Albert-Ludwigs-Universität Freiburg, Freiburg, Germany; 2 Department of Clinical Psychology, VU University Amsterdam, Amsterdam, The Netherlands; 3 Eindhoven University of Technology, Eindhoven, The Netherlands; 4 Royal Netherlands Academy for Arts and Sciences, Utrecht University, Utrecht, The Netherlands; 5 Koç University, Istanbul, Turkey; University of Leicester, United Kingdom

## Abstract

Recent work suggests that people evaluate target stimuli within short and flexible time periods called evaluation windows. Stimuli that briefly precede a target (forward primes) or briefly succeed a target (backward primes) are often included in the target's evaluation. In this article, the authors propose that predictable forward primes act as “go” signals that prepare target processing, such that earlier forward primes pull the evaluation windows forward in time. Earlier forward primes may thus reduce the impact of backward primes. This shifting evaluation windows hypothesis was tested in two experiments using an evaluative decision task with predictable (vs. unpredictable) forward and backward primes. As expected, a longer time interval between a predictable forward prime and a target eliminated backward priming. In contrast, the time interval between an unpredictable forward primes and a target had no effects on backward priming. These findings suggest that predictable features of dynamic stimuli can shape target extraction by determining which information is included (or excluded) in rapid evaluation processes.

## Introduction

In their daily lives, people are exposed to a kaleidoscope of objects and events. Because each of these objects or events may signal potential threats or opportunities, people need to be able to quickly extract the evaluative meaning from the continuous stream of stimuli. For instance, in conversations, people may have to process rapid changes in our conversation partner's facial expression. Likewise, in traffic, people may often need to quickly monitor specific states of traffic lights to take appropriate actions. Recent work suggests that people evaluate such target stimuli within short and flexible time periods that have become known as “evaluation windows” [1], [2]. Evaluation windows play a key role in social evaluation processes by determining which evaluative information is integrated into a given target evaluation. It is therefore important to learn which factors shape how and when perceivers set evaluation windows.

In predictable environments, certain cues may reliably signal the onset of a target stimulus. For instance, when you tell your partner a joke, the punch line will usually precede any spontaneous reaction of your partner to that joke. Such a predictable environment may be simulated in an experimental setting, in which some irrelevant stimulus may consistently precede a target object [3], [4]. Recent research suggests that people may use such predictable stimuli preceding a target stimulus as go signal primes to initiate preparatory, target-relevant processes, which allow for overall faster target responses [1], [2]. Although this preparatory function of go signal primes on target responding is currently well-established [1], [2], it remains an open question what kind of target-relevant processes are facilitated by go signal primes.

**Figure 1:**
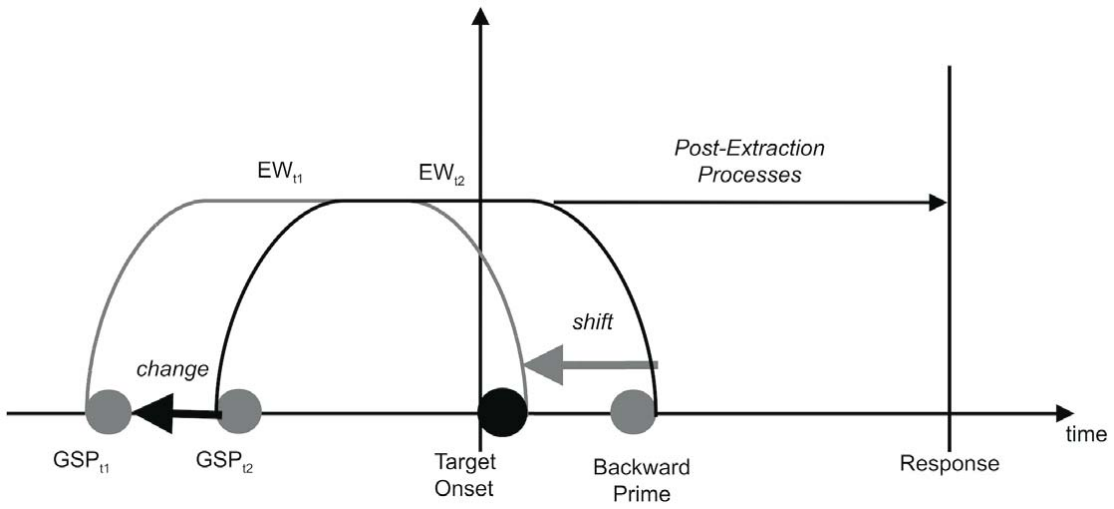
The Shifting Evaluation Window Hypothesis. An earlier go signal prime (GSP_t1_) shifts the evaluation window forward (dotted line) compared to a later go signal prime (GSP_t2_). Due to this shift, the backward prime is excluded from the evaluation window.

In the present paper, we propose that go signal primes facilitate the immediate extraction of target information from a stimulus stream. Accordingly, an “early” go signal prime, which precedes a target at a somewhat longer time-interval, may pull the entire target extraction period (i.e. the *evaluation window*) somewhat forward in time (and vice versa for “late” go signal primes). We refer to this as the *shifting evaluation windows* (SEW) hypothesis. The shifting evaluation hypothesis has important implications for the kinds of information that people include in their evaluative judgments. Research on so-called backward priming has shown that stimuli that briefly succeed a target (or *backward primes*) are often included in target evaluation, even when people are explicitly told to ignore such stimuli [5]. If the evaluation window shifts forward in time, then backward primes will have a reduced opportunity to influence people's evaluations. Consequently, the shifting evaluations hypothesis predicts that early onset of go signal primes will lead to a reduction of the impact of subsequent backward primes (see Figure 1).

In what follows, we briefly review the relevant evaluative priming literature. Next, we more fully discuss how the evaluation window account [1], [2] provides the theoretical basis for the present research. Finally, we present two studies that tested the SEW hypothesis in a modified evaluative priming paradigm (cf. [3]).

### Dynamics of Rapid Evaluations

Over the last two-and-a-half decades, rapid evaluation processes have been the focus of intense scientific scrutiny within the evaluative priming literature [6]–[8]. Most of this research has used the so-called evaluative decision task. In this task, participants are briefly exposed to positive and negative primes and targets, and must quickly and accurately indicate the target's valence (e.g., [9]). Participant's responses to the target are typically faster and/or more accurate, if the targets are preceded by a prime of the same (vs. different) valence. Such *evaluative priming effects* reliably occur at brief stimulus onset asynchronies (i.e. SOAs; [6], [7]) between primes and targets. By now, evaluative priming effects have been obtained with numerous stimuli such as words, pictures, sounds, and even odors; and across different target processes, such as target evaluation, target naming, and lexical decision tasks [7], [10]. Although evaluative priming research has typically used forward primes, which precede target presentation, evaluative priming also occurs with primes that occur briefly after target presentation, called backward primes [5]. Consequently, the time window within which people extract evaluative information from the stimulus stream appears to start somewhat before and end somewhat after target presentation [1], [5].

Evaluative priming is subject to a number of moderating factors. For instance, evaluative priming effects are largely restricted to contexts that render the evaluative stimulus dimension relevant [11] or at least salient [12]–[15]. Furthermore, people appear, at least to some degree, capable of intentionally regulating the influence of evaluative primes on target responding (e.g., [16]–[19]). Finally, recent priming research with multiple sequential primes suggests that multiple stimuli may interact in complex ways ([3], [20], but see 1 for contrary findings). Overall, research on evaluative priming suggests that the size and direction of evaluative priming effects varies dynamically, in response to people's task goals, perceptual factors, and the context of other evaluative stimuli.

The evaluative priming literature is large, consisting of hundreds of experiments, and complex, due to a sizable number of moderating variables (see [1], [7]). Consequently, it is challenging to develop an integrative process model of evaluative priming effects. Nevertheless, Klauer and colleagues [1] rose to this challenge by proposing an *evaluation window account*, a powerful and highly generative approach that integrates many findings of the evaluative priming literature. A comprehensive discussion of the evaluation window account can be found elsewhere [1]. We suffice with a general outline and discuss only those details that are directly relevant within the present context.

### The Evaluation Window Account

The evaluation window account [1] proposes that people extract target evaluations from the incoming stream of stimulus activation within short evaluation windows. Within evaluation windows, people monitor incoming stimulus activation for changes using a positive and a negative counter. Because target extraction from a stream of stimuli is somewhat imperfect and the counters do not differentiate between prime and target activation, temporally proximal primes are included in the evaluation window and yield evaluative priming effects. In contrast, temporally more distal forward primes are excluded from the evaluation window. Moreover, temporally more distal primes are assumed to activate the relevant evaluative counter. Because this activation should hinder the detection of changes within the respective counter (cf. Weber Fechner Law, see [1]), distal forward primes are expected to elicit reversed priming effects.

The evaluative window account can accommodate the majority of known findings within the evaluative priming literature [1]. Moreover, the evaluative window account affords a number of new empirical predictions. Most relevant here, the evaluation window account proposes that people exert a level of control in determining when they set up the evaluation window. For instance, when people are presented with predictable stimulus sequences, they may intuitively use the last prime before the target (i.e. the last forward prime) as a go signal to set up the evaluation window before target onset. This signal-prime mechanism fits with findings that overall response time decreases with longer SOAs (e.g., [1], [5]), given that longer SOAs provide participants with more time to prepare for target onset. Notably, the evaluation window account assumes that synchronization between go signal prime and the onset of the evaluation window breaks down at higher SOAs. This prediction fits with the typical dissolution of evaluative priming effects at longer SOAs (above 300 ms, see [7]). Although the evaluation window account [1], [2] is a major step forward, important questions remain. For one thing, it is unclear what happens to the evaluation window once it has been set up by the go signal prime. The evaluation window account assumes that signal primes “trigger some amount of preparatory target-relevant processing” [1], which explains the general findings of overall decreases in response time with longer prime-target intervals (i.e. more preparation time). But which part of the target evaluation process is facilitated by a go signal prime?

### The Shifting Evaluation Window Hypothesis

Building upon and extending the evaluation window account [1], we suggest that the go signal prime does not only facilitate the opening of a new evaluation window, but also facilitates the early closure of the evaluation window. In effect, the go signal prime moves, depending on its temporal proximity to the target, the entire evaluation window forward (or backward) in time. We therefore refer to this notion as the *shifting evaluation window* (SEW) hypothesis, which is graphically displayed in Figure 1.

To unpack the SEW hypothesis, it is useful to think of it as having an old (empirically established) part and a new (as yet untested) part. The old part of the SEW hypothesis refers to the facilitating effects of the go signal prime on the opening of the evaluation window. As we already discussed, there is already initial evidence for this effect [1], [2]. In terms of the evaluation window account [1], the go signal prime opens the evaluation window by leading perceivers to start monitoring the environment for any changes in the positive and negative counters. Go signal primes that are congruent with the target provide a longer burst of activation in the correct counter, if the signal prime precedes the target a somewhat longer time interval. Conversely, go signal primes that are incongruent with the target result in less simultaneous co-activation with both counters, if the signal prime precedes the target at a somewhat longer time interval. At this point, we arrive at the new part of the SEW hypothesis. Once perceivers detect a clear change in the positive or negative counters, perceivers are likely to infer (implicitly) that they have extracted the evaluative targets valence. Thus, a somewhat earlier onset of the go signal prime will prompt perceivers to close the evaluation window somewhat earlier. In sum, earlier go signal primes result in an overall temporal shift in the evaluation window, leading to earlier onset and offset of the timeframe during which environmental stimulus information is evaluated.

The SEW hypothesis allows for one important new empirical prediction, which relates to the interplay between forward evaluative primes (i.e. primes presented before target onset) and backward evaluative primes (i.e. primes presented after target onset). Specifically, the SEW hypothesis predicts that, under certain conditions, forward primes may eliminate the impact of subsequent backward primes. Specifically, forward primes may eliminate backward priming effects when forward primes are predictable (which permits perceivers to use them as go signals) and if they occur at longer intervals before the target (leading to a greater temporal shift of the evaluation window). Because earlier (rather than later) forward primes allow perceivers to prepare more for the onset of the target, earlier forward primes should further reduce overall response times.

Although we regard the SEW hypothesis as plausible, we note that its rationale is based on the validity of the evaluation window account. If early go signal primes reduce overall response times through a different kind of mechanism, other empirical predictions are possible. For instance, one could propose that go signal primes may trigger some post-extraction process, such as conflict resolution (cf. [3]) or some later decision-related or response-related process [21]–[23]. From this perspective, early signal primes might facilitate the separation of prime and target information, *after* initial stimulus extraction. Such post-extraction processes could also account for overall faster response times (cf. [3]). However, if go signal primes would exclusively prepare conflict-resolution processes that occur *after* stimulus extraction, the timing of go signal primes should not alter the closure of the extraction period (and thus backward priming) Thus, whether an early go signal prime facilitates the end of stimulus extraction processes remains an empirical question.

### Present Research and Hypotheses

We designed the present research to test the SEW hypothesis, or the idea that an earlier (rather than later) onset of a forward prime could lead to a forward shift of the evaluation window. To this end, we designed two experiments that used a variation of the evaluative decision task with both forward and backward evaluative primes [3]. Forward evaluative primes, which preceded the target, served as potential go signal primes. Backward evaluative primes always succeeded the target. The emergence of a backward priming effect was therefore an index of whether and how the forward (signal) primes influenced the end of the stimulus extraction process (i.e. closure of the evaluation window; cf. [24]).

According to the SEW hypothesis, early forward primes pull evaluation windows forward in time by enabling preparatory processing of the target stimulus (see Figure 1). From the literature on task preparation [25]–[27], it is known that having more time allows for more effective preparatory processing. Accordingly, we manipulated the stimulus onset asynchrony (SOA) between primes and target in both experiments. Because both forward and backward priming effects are generally short-lived [3], [5], we used relatively short SOAs of 150 ms and 250 ms before and after target onset.

In line with the SEW hypothesis, we predicted a stronger influence of the forward primes (i.e. go signal primes) on shifting the evaluation windows at longer than at shorter SOAs. Consequently, we predicted that longer SOAs of forward primes would result in a reduction in backward priming effects relative to shorter forward SOAs. In contrast, if early forward primes merely facilitate stimulus conflict resolution processes that occur after stimulus extraction, the SOA of the forward primes should not affect backward priming effects. We tested these predictions in Experiments 1 and 2. Experiment 2 further manipulated the predictability of the SOAs, a crucial variable that should moderate the ability of forward primes to act as go signals (see [1]). In line with prior research, we expected that forward primes would only be used as go signals within predictable stimulus sequences. In line with the SEW hypothesis, we predicted that the SOA of the forward primes would only moderate backward priming (i.e. Backward Congruency) when forward primes preceded target within predictable time intervals (i.e. constant per block) but not unpredictable time intervals [1].

## Experiment 1

### Method

#### Participants and design

Sixty paid volunteers (42 women, 18 men, average age = 21 years) participated at the VU University Amsterdam. All procedures were executed in compliance with relevant laws and institutional guidelines and were approved by the ethics committee of the Faculty of the VU University Amsterdam. All participants gave written informed consent for their participation. The experiment had a 2(Forward SOA: 150/250 ms)×2(Backward SOA: −150/−250 ms)×2(Forward Congruency)×2(Backward Congruency) within-subject design.

#### Materials

Practice stimuli consisted of 12 positive and 12 negative adjectives; the experimental stimuli consisted of another set of 48 positive (e.g., *pleasant, friendly, clean*) and 48 negative adjectives (e.g., *alone, illegal, dishonest*). All stimuli were taken from the same pilot-tested stimulus pool as Fockenberg et al. [5]. Positive and negative adjectives differed qua valence (1 = negative to 9 = positive; *t*(1,94) = 90.59, *p*<.001, *M* = 7.7 vs. *M* = 2.5), but not word length, *t*(1,94) = 1.12, *p* = .27, *M* = 7.4 vs. *M* = 6.8. Primes were presented in black and targets in red against a white background.

#### Procedure

Participant were informed that they would be repeatedly exposed to three words in close succession, and that their task was to indicate by keystroke (“a”, “l”) as fast and accurate as possible whether the second, red word was positive or negative. Participants then received 8 practice trials (SOA ±200 ms), in which each valence combination was presented once in random order. During practice trials participants received feedback and could repeat the practice trials, if they wanted. Afterwards, participants completed an evaluative decision task, in which no feedback was given and which consisted of 4 blocks of 32 trials. Each block had different Forward and Backward SOAs (i.e. +150/−150 ms, +150/−250 ms, +250/−150 ms, +250/−250 ms). The order of blocks was randomized. Participants could take a short break between blocks. Within blocks, all stimuli were randomly allocated to the 16 cells of the design in each block and trials were run in random order.

The sequence of events in each trial was as follows: First, a fixation cue (***) was presented for two seconds and replaced by the first prime (i.e. the forward prime), which remained on the screen for 100 ms. The target was then displayed either 50 ms or 150 ms after prime offset, depending on the block (Forward SOA = 150 ms vs. 250 ms). The target remained on the screen for 100 ms and was replaced by the second prime (i.e. the backward prime) either 50 ms or 150 ms after target offset, depending on the block (Backward SOA = −150 ms vs. −250 ms). The second prime remained on the screen for 100 ms, and was then replaced by a blank screen until participants responded. After participants responded, the next trial was immediately initiated.

### Results and Discussion

One participant was excluded because of extremely high error rates (ER, according to Tukey's criterion [28]). Response times (RTs) of incorrect responses and below (<.01%) or above (2.4%) 2.5 standard deviations (*SD*s) from the individual average RTs were excluded from analyses. Average RT was 650 ms (*SD* = 90.56), average ER was 8.7% (*SD* = 0.06). In both experiments, backward priming effects were only significantly moderated in the RTs (cf. [3]). Therefore we focus on the RTs. The results for ERs can be found in the Supporting Information S1.

The analysis revealed a main effect of Forward SOA and Backward SOA, *F*(1,58) = 16.63, *p*<.001, η_p_ = .22, and *F*(1,58) = 10.97, *p* = .002, η_p_ = .16, respectively, reflecting overall slower responses when primes occurred at ±150 ms compared to ±250 ms. This slow-down with shorter SOAs has been consistently observed in the literature and likely reflects preparatory processes that are initiated by the signal prime [1].

The analysis furthermore revealed a main effect of Forward Congruency, *F*(1,58) = 5.67, *p*<.05, η_p_ = .09, reflecting overall faster responses after congruent than incongruent forward primes (*M* = 646 ms, *SD* = 12.47, vs. *M* = 656 ms, *SD* = 11.41), a forward priming effect). Likewise, the analysis revealed a main effect for Backward Congruency, *F*(1,58) = 5.96, *p*<.05, η_p_ = .09, indexing overall faster responses after a congruent than incongruent backward prime (*M* = 646 ms, *SD* = 11.41, vs. 655 ms, *SD* = 12.40), a backward priming effect). The backward priming effect was qualified by a marginally significant Backward SOA×Backward Congruency interaction, *F*(1,58) = 3.94, *p* = .052, η_p_ = . 06. As revealed by planned contrasts, backward priming only occurred at a Backward SOA of −150 ms, (*M* = 651 ms, *SD* = 10.90, vs. *M* = 668 ms, *SD* = 13.08), *F*(1,58) = 7.13, *p* = . 01, η_p_ = .11; but not −250 ms (*M* = 641 ms, *SD* = 12.82, vs. *M* = 643 ms, *SD* = 12.42), *F*(1,58)<1.

**Figure 2:**
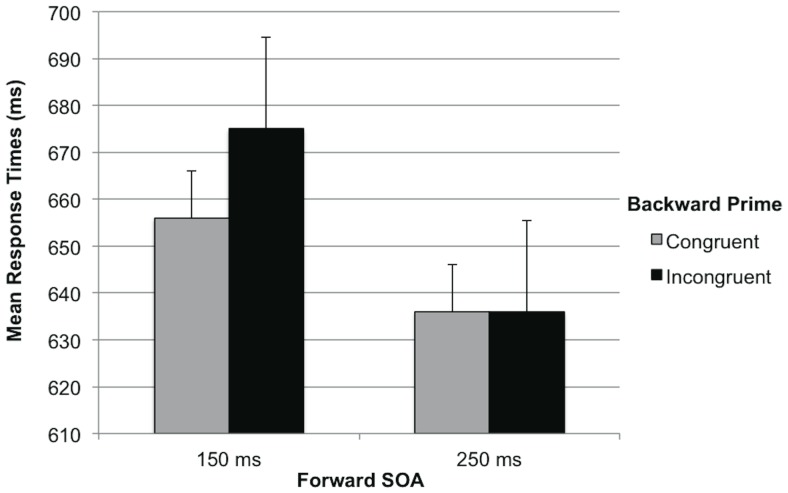
Response Time as a function of Forward SOA and Backward Congruency (Experiment 1).

Importantly, we found the Forward SOA×Backward Congruency interaction predicted by the SEW hypothesis, *F*(1,58) = 10.92, *p* = .002, η_p_ = .16 (see Figure 2). Participants only responded faster after congruent than incongruent backward primes when the forward prime preceded the target by 150 ms, *F*(1,58) = 11.53, *p* = .001, η_p_ = .17. When the forward prime preceded the target by 250 ms, no backward priming occurred, *F*(1,58)<1. That is, in line with the SEW hypothesis, an earlier onset of the forward prime was accompanied by an earlier offset of backward priming. No other effects reached statistical significance, *F*s(1,58)≤2.34, *p*s≥.13.

The results of Experiment 1 provide clear support for the SEW hypothesis: When forward primes preceded the target at a short SOA, backward priming occurred; when forward primes preceded the target at a longer SOA, no backward priming was found. This suggests that forward prime impacted the target extraction process, such that an earlier forward prime allowed people to stop stimulus extraction earlier than a later forward prime.

## Experiment 2

Although Experiment 1 provided initial support for the SEW hypotheses, a critical assumption of the evaluation window account [1] regarding the “go” signal function of forward primes remained untested. Specifically, the evaluation window account assumes that people only use the last forward prime as a “go” signal when it provides a predictable signal for target onset [1]. In Experiment 1, this was always the case because forward primes consistently preceded targets at a specific, fixed time interval within each block (i.e. blockwise SOA manipulation). However, if the last forward prime precedes the target at unpredictable, variable time intervals (e.g., trialwise SOA manipulation), people can no longer use the forward prime as an effective “go” signal. When the timing of forward primes is unpredictable, the onset of the evaluation window can no longer be synchronized with the forward prime [1]. As a result, the placing of the evaluation window will become more erratic and less systematic. In brief, the last forward prime should only be able to shift the evaluation windows, if the forward prime proceeds the target at predictable, but not unpredictable time intervals. We tested this extension of the SEW hypothesis in Experiment 2.

### Method

#### Participants and design

Sixty paid volunteers (42 women, 17 men, average age = 21 year; on participant did not provide demographic data) participated at the Albert-Ludwigs-Universität Freiburg. All procedures were performed in compliance with relevant laws and institutional guidelines and were approved by the ethics committee of the Psychology department of the Albert-Ludwigs-Universität Freiburg. All participants gave written informed consent for their participation. The experiment had a 2 (SOA Variation: blockwise/trialwise)×2(Forward SOA: 150/250 ms)×2(Backward SOA: −150/−250 ms)×2(Forward Congruency)×2(Backward Congruency) within-subject design.

#### Materials

The Dutch adjectives were replaced by 48 positive and 48 negative German adjectives. As indicated by pilot tests, positive and negative adjectives differed in valence (−3 = negative to +3 = positive; *t*(1,94) = 33.57, *p*<.001, *M* = 1.67 vs. *M* = −1.82), but not word length (both *M*s = 6.77 letters). Also, participants now responded via the interior keys of two computer mice positioned to the left and right [29].

#### Procedure

The procedure was identical to that of Experiment 1, except that participants completed an additional 4 blocks of 32 trials in which Forward SOA and Backward SOA varied randomly per trial. Consequently, participants were presented with 8 blocks of 32 trials. The 4 blocks with blockwise SOA variation and the 4 blocks with trialwise SOA variation were run in sequential order. The order of these units was counterbalanced between participants.

### Results and Discussion

One participant did not follow instructions, as evidenced by an extremely high ER of 93.4%; another participant was an extreme outlier in the error rates (ERs) according to Tukey's criterion [28]. Both participants were excluded from the analyses. Response times of incorrect responses and below (<.01%) or above (2.7%) 2.5 standard deviations from the individual average RTs were excluded from analyses. The average RT was 631 ms (*SD* = 160.52), the average ER was 9.8% (*SD* = 0.07).

Average RTs were subjected to a 2(SOA Variation: Blockwise/Trialwise)×2(Forward SOA: 150/250 ms)×2(Backward SOA: −150/−250 ms)×2(Forward Congruency)×2(Backward Congruency) repeated measures ANOVA. This analysis revealed a significant interaction between all five variables, *F*(1,57) = 4.35, *p*<.05, η_p_ = .07. To disentangle this interaction, we repeated this analysis for blockwise and trialwise SOA Variation separately.

#### Blockwise SOA-Variation

The analysis revealed a marginally significant main effect for Forward SOA *F*(1,57) = 3.36, *p* = .07, η_p_ = .06, indicating slower responses when the forward prime preceded the target by 150 ms (*M* = 644 ms, *SD* = 26.19) than by 250 ms (*M* = 627 ms, *SD* = 24.00). The analysis also revealed a significant Forward Congruency effect, *F*(1,57) = 4.30, *p*<.05, η_p_ = .07, that indicated faster responses after congruent than incongruent forward primes across SOAs (*M* = 630 ms, SD = 26.05, vs. *M* = 641 ms, SD = 23.56), reflecting a forward priming effect. Furthermore, a marginally significant Backward Congruency effect occurred, *F*(1,57) = 3.18, *p* = .08, η_p_ = .05, indicating faster responses when the target was succeeded by congruent than incongruent backward primes (*M* = 631 ms, *SD* = 23.12, vs. *M* = 640 ms, *SD* = 26.45), reflecting a backward priming effect.

**Figure 3:**
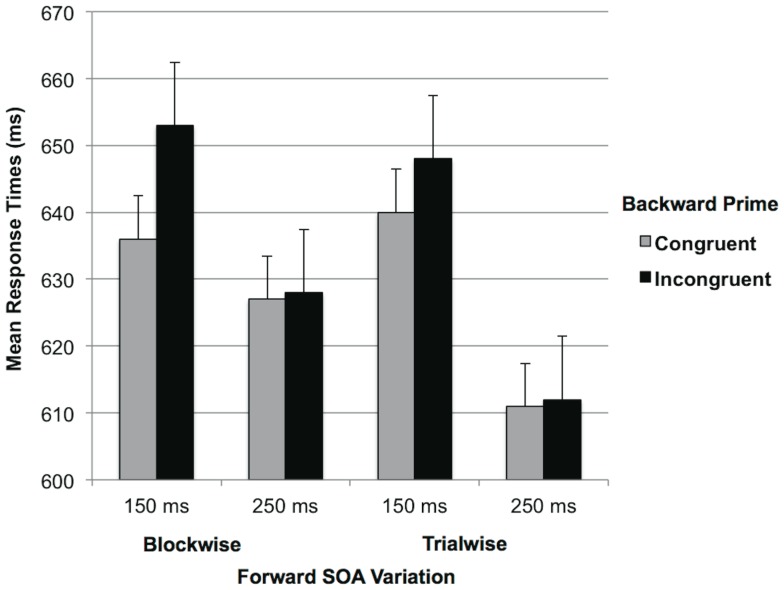
Response Time as a function of SOA Variation, Forward SOA and Backward Congruency (Experiment 2).

Importantly, we replicated the predicted Forward SOA×Backward Congruency interaction from Experiment 1, *F*(1,57) = 4.69, *p*<.05, η_p_ = .08 (see Figure 3). As predicted by the SEW hypothesis, participants only responded faster after congruent than incongruent backward primes, when the forward prime preceded the target by 150 ms (*M* = 636 ms, *SD* = 24.11, vs. *M* = 653 ms, *SD* = 28.53), *F*(1,57) = 6.20, *p*<.05, η_p_ = .10. When the forward prime preceded the target by 250 ms, no backward priming occurred (*M* = 627 ms, *SD* = 23.28, vs. M = 628 ms, *SD* = 25.03), *F*(1,56)<1.

Notably, the analysis also revealed a Backward SOA×Forward Congruency×Backward Congruency interaction, *F*(1,57) = 5.62, *p*<.05, η_p_ = .09. This interaction replicated the congruency-based moderation observed by Fockenberg et al. [3], who attribute this effect to an increase in cognitive control that is triggered by the response conflict between forward prime and target. Specifically, at short backward SOAs (−150 ms), planned contrasts revealed only backward priming effects when the forward prime was congruent with the target (*M* = 626 ms, *SD* = 23.67, vs. *M* = 646 ms, *SD* = 28.42), *F*(1,57) = 5.42, *p*<.05, η_p_ = .09, but not when the forward prime was incongruent with the target (*M* = 646 ms, *SD* = 24.27, vs. *M* = 643 ms, *SD* = 21.52), *F*(1,57)<1. At longer Backward SOAs (−250 ms), no backward priming occurred, irrespective of whether the forward prime was congruent (*M* = 623 ms, *SD* = 24.34, vs. *M* = 627 ms, *SD* = 29.88), *F*(1,57)<1, or incongruent (*M* = 629 ms, *SD* = 22.49, vs. *M* = 645 ms, *SD* = 28.32), *F*(1,57) = 2.62, *p* = .11, with the target. No other effects reached statistical significance, *F*s(1,57)≤2.31, *p*s≥.14.

In line with the SEW hypothesis, and replicating Experiment 1, an earlier onset of the forward prime (i.e. the go signal) was accompanied by an earlier offset of backward priming. Notably, the SEW pattern was not only replicated for the blockwise trials of the whole participant sample, but also for the blockwise trials of the subset of the participants who received the blockwise SOA variation (and thus, to that point, followed the same procedure as Experiment 1, see Supporting Information S2). Unlike Experiment 1, Experiment 2 furthermore provided evidence for a significant moderation of backward priming due the congruency between the forward prime and the target (cf. [3]). That is, backward priming effects were eliminated, if the forward prime was incongruent with the target, which may reflect the working of conflict-induced correction processes [3]. Notably, although Experiment 1 did not yield this interaction, theory-driven contrasts revealed the same pattern as in the current experiment (see Supporting Information S3). Because neither study provided evidence for a moderation of this interaction by the timing of the forward prime, the conflict-based effects appear independent of the go signal prime-induced shifts of the evaluation windows.

#### Trialwise SOA-Variation

The analysis revealed a significant main effect of Forward SOA, *F*(1,57) = 65.98, *p*<. 001, η_p_ = .54, indicating that participants responded more slowly when the forward prime preceded the target at 150 ms (*M* = 644 ms, *SD* = 19.19) than 250 ms (*M* = 611 ms, *SD* = 17.76). Likewise, the analysis revealed a main effect of Backward SOA, *F*(1,57) = 8.27, *p* = .006, η_p_ = .13, indicating participants responded slower, when the target was succeeded by a backward prime by 150 ms (*M* = 634 ms, *SD* = 19.42) than 250 ms (*M* = 621 ms, *SD* = 17.57). Both main effects were qualified by a significant Forward SOA×Backward SOA interaction, *F*(1,57) = 4.61, *p*<.05, η_p_ = .08, indicating that participants responded most slowly when both forward and backward primes occurred at ±150 ms (*M* = 654 ms, *SD* = 20.69), faster when the forward prime occurred at 150 ms and the backward prime at 250 ms (*M* = 634 ms, *SD* = 18.11), *F*(1,57) = 11.03, *p* = .002, η_p_ = .16, and fastest when the forward prime occurred at 250 ms and the backward prime occurred at an SOA of −150 ms (*M* = 614 ms, SD = 18.51), *F*s(1,57)≥12.57, *p*≤.001, η_p_ = .18, or both primes occurred at an SOA of ±250 ms (*M* = 608 ms, *SD* = 17.40), *F*s(1,57)≥22.92, *p*<.001, η_p_ = .29. The last two conditions did not differ significantly, however, *F*(1,57)<1.

Importantly, the analysis furthermore only revealed a significant main effect of Forward Congruency, *F*(1,57) = 4.41, *p*<.05, η_p_ = .07, which indicated faster responses after congruent than incongruent forward primes (*M* = 623 ms, *SD* = 19.27, vs. *M* = 632 ms, *SD* = 17.73), a forward priming effect). No other effects reached statistical significance, *F*(1,57)≤2.66, *p*≥.11. Specifically, in line with the evaluation window account, the analysis revealed no Forward SOA×Backward Congruency interaction, *F*(1,57)<1 (see Figure 3, for an illustration). That is, when the forward prime formed no predictable indicator for target onset (or “go” signal), its temporal location had no influence on backward priming, and thus the inclusion of stimuli that occur after target onset within the target's evaluation window. Also, the analysis yielded no evidence for a moderation of backward priming through the congruency of forward prime and the target (see also Supporting Information S3).

Also of interest, the analysis also did not reveal any general effect of Backward Congruency, *F*(1,57) = 1.31, *p* = .31. This lack of backward priming might be due to a general increase in cognitive control during unpredictable primes (cf. [30]). Alternatively, the need for quick responses and the unpredictability of the target may have led participants to generally start the monitoring process somewhat earlier, therefore shifting the evaluation window somewhat forward in general. As forward priming did not vary across Forward SOAs, this explanation cannot be explanation cannot be excluded. Further research may be needed to validate and disentangle this phenomenon.

## General Discussion

In the present article, we tested the novel hypothesis that an earlier (rather than later) onset of a forward prime could lead to a forward shift of the evaluation window. In line with this shifting evaluation window (SEW) hypothesis, the results of two experiments showed that the SOA of a forward prime moderated the impact of subsequent backward primes on target evaluations. Specifically, backward evaluative priming effects were eliminated with longer time intervals between the forward prime and the target. Furthermore, in line with the evaluation window account [1], [2], this moderation did not occur when the use of forward prime's as go signals was eliminated due to the unpredictability of the onset of the prime in relation to the target (Experiment 2).

The present research is the first to test the implications of the “go” signal assumption before and after target onset. By so doing, our findings provide further support for the assumption that people use the last forward prime as a go signal that prepares target processing in predictable stimulus environments [1], [2]. Additionally, these results provide new insights concerning the nature of these preparation processes: The signal prime appears to directly facilitate the stimulus extraction process (e.g., evaluation window [1]), instead of merely later, separate processes such as a post-extraction conflict resolution (cf. [3]). As a consequence, an earlier onset of evaluation windows in Experiments 1 and 2 seems to have allowed people to close off the evaluation windows earlier. As such, it appears that the signal prime serves to anchor the overall evaluation window in time.

The evaluation window account proposes that the last forward prime loses its utility as a go signal if it does not precede the target at predictable, short time intervals [1]. Consistent with this, when the forward primes preceded targets at unpredictable, variable times in Experiment 2, the temporal location of forward primes did not moderate the offset of stimulus extraction anymore. Instead, only a general forward priming effect emerged, irrespective of stimulus SOAs. This suggests that when participants were exposed to unpredictable stimulus sequences, they used narrower evaluation windows (i.e. no backward primes), which ended briefly after target onset before any backward primes could influence target processing.

Both of the present experiments also provided some evidence for the influence of congruency between forward prime and target on backward priming, which was also observed in previous research [3]. Specifically, incongruent forward primes yielded a reduced influence of backward primes, which could reflect an increase in conflict-resolution processes that hinder backward prime integration [3]. Whereas this pattern only emerged in theory-driven a priori contrasts in Experiment 1, the moderation was statistically significant in Experiment 2 (for more details, see Supporting Information S1). In both studies, this pattern was restricted to backward primes that occurred very briefly after the target. This is in line with the notion that backward priming effects are more short-lived than forward priming effects [5]. Furthermore, this effect was only found for predictable forward primes, which may serve as go signal prime. However, it appeared to be independent of the timing of go signal primes. The congruency-based moderation of backward priming effects is not expected by the evaluation window account. Given the somewhat weak congruency-based moderation, however, further research may be needed for any firm conclusions concerning this effect.

Overall, the present research further highlights the importance of the prime that immediately precedes the target in priming tasks with multiple evaluative primes [1], [3], [20]. Specifically, within predictable environments, this prime does not only contribute relevant evaluative information to the evaluation process, but also works as a gatekeeper that determines what evaluative information is included in the evaluation process. Whereas previous research suggests that the congruency of this prime and the target may determine the influence of previous forward and subsequent backward primes [5], the present research adds that the temporal distance between this prime and the target also moderates the influence of backward primes, specifically in predictable stimulus streams (cf. [1]).

An important methodological implication of the present work is that, by presenting predictable cues before target onset, researchers may to some degree determine when participants will assess target information. As already noted by Klauer et al. [1], “any factor that is likely to affect the positioning of the evaluation window has a direct influence on the size and potentially the sign of evaluative priming effects.” (p. 282). As the present research testifies, the presentation of an early or late signal cue may have downstream consequences for how later stimuli are processed. This may be relevant, for example, in paradigms that apply backward masks to restrict target processing [31], [32]. The present findings suggest that with shorter time intervals between forward primes and targets, backward masks may affect target extraction to a greater degree, because participants would need more time to extract the target. As a result, the prime may have a greater influence on target responses, resulting in stronger priming effects. Notably, it remains an open question whether “go” signals have similar effects as in the present research, if targets do not require explicit evaluative responses (e.g., in a naming task, see 4,7]. However, previous research suggests that similar effects may be expected for these kinds of tasks, provided that the evaluative dimension is sufficiently salient ([13], [14], see also [1], page 283).

Extrapolating to everyday contexts, the present findings may reflect the tendency for perceivers to not passively await the occurrence of a dynamic target object to initiate their evaluation, but rather initiate the process in anticipation of the proper moment. For instance, a car driver may use his experience of how long it takes to stop his car to predict the best moment to respond to the traffic lights. Likewise, people may monitor and evaluate changes in facial expressions of another person at specific moments, to determine their emotional response to some event. At the moment, we still know relatively little about how people spontaneously set such internal cues to time their basic evaluation and categorization processes within dynamic environments. As suggested by the present research, both the timing and congruency of what people perceive may jointly shape how they evaluate environmental objects.

## Supporting Information

Supporting Information S1 **Results of the analyses of the Error Rates for** **Experiment 1** **and 2.**
(RTF)

Supporting Information S2 **Experiment 2** **: Replication of Shifting Evaluation Window effect for Blockwise Condition for participants, who received this condition first.**
(RTF)

Supporting Information S3 **Additional Analysis: The Moderating Role of Congruency between Go Signal Prime and Target [cf. 3] for** **Experiment 1** **and 2.**
(RTF)
