# Supplementary material for: Shifting Evaluation Windows: Predictable Forward Primes with Long SOAs Eliminate the Impact of Backward Primes
Source: PLoS One. 2013 Jan 24;8(1):e54739. doi: 10.1371/journal.pone.0054739 (PMC3554650; doi:10.1371/journal.pone.0054739)
Supplement: Supporting Information S1 — Results of the analyses of the Error Rates for Experiment 1 and 2. (RTF) [file pone.0054739.s001.rtf]

Supporting Information S1
 Experiment 1, Error Rates
	The analysis revealed a main effect of Backward Congruency, F(1,58) = 5.26, p <.05, ηp = .08 (M = 8.0 % vs. M = 9.4 %), and a Forward SOA x Backward SOA interaction, F(1,58) = 7.10, p = .01, ηp = .11, reflecting most errors at an SOA of ± 150 ms (M = 10.1 %) and fewer at +150/-250 ms (M = 8.1 %)  and 250/-150 ms  (M = 7.8 %),  Fs(1,58) ≤ 4.84, p <. 05, ηp ≥ .08. At ±250 ms, the error rate fell somewhat in between (M = 8.8 %), and did not differ significantly from the others, Fs(1,58) ≤ 2.29, p ≥ .14. No other effects reached statistical significance, Fs(1,58) ≤ 1.87, ps ≥ .18.
Experiment 2, Error Rates
	This analysis revealed no significant differences between blockwise and trialwise SOA Variation, Fs(1,57) ≤ 2.13, ps ≥ .15. Therefore, no separate analyses for both were conducted and the results will be reported across both conditions. The analysis revealed a main effect of Forward SOA, F(1,57) = 10.63, p = .002, ηp = .16, and Backward SOA, F(1,57) = 6.45, p < .05, ηp = .10, reflecting more errors at temporally close forward primes (M = 10.5 % vs. M = 9.0 %), and backward primes (M = 10.4 % vs. M = 9.1 %). Both main effects were qualified by a Forward SOA x Backward SOA interaction, F(1,57) = 6.99, p <.05, ηp = .11, reflecting more errors at an SOA of ± 150 ms (M = 11.7 %) than at any other SOA combination (M150/-250 = 9.3 % or M250/-150 = 9.1 % or M±250 = 8.9 %), Fs(1,57) ≥ 14.08, ps < .001, ηp ≥ .20. Except for an SOA of  ± 150 ms , all SOAs yielded a similar amount of errors, F(1,57) < 1.
	Furthermore, the analysis revealed a main effect for Forward Congruency, F(1,57) = 5.93, p <.05, ηp = .09, reflecting less errors after congruent than incongruent forward primes (M = 9.0 %  vs. M = 10.5 %, i.e. forward priming). This main effect was qualified by a Forward SOA x Forward Congruency interaction, F(1,57) = 4.68, p <.05, ηp = .08, a marginally significant BSOA x Forward Congruency interaction, F(1,57) = 3.46, p = .07, ηp = .06, and a significant FSOA x BSOA x Forward congruency interaction, F(1,57) = 7.07, p = .01, ηp =.11. As indicated by planned contrasts, forward priming effects only occurred, when forward and backward primes occurred at an SOA of ± 150 ms (M = 9.4 % vs. M = 14.1 %), F(1,57) = 26.38, p <.001, ηp =.32. In contrast, no forward priming occurred at SOAs of 150/-250 (M = 9.1 % vs. M = 9.6 %), 250/-150 ms (M = 8.9 % vs. M = 9.2 %) or ±250 ms (M = 8.5% vs. M = 9.3 %), all Fs(1,57) < 1.
	Likewise, the analysis revealed a main effect for Backward Congruency, F(1,57) = 11.10, p = .002, ηp = .16, reflecting less errors when the target was succeeded by a congruent than incongruent backward prime (M = 9.1 % vs. 10.4 %, i.e. backward priming). This main effect was qualified by a marginally significant Backward SOA x Backward Congruency interaction, F(1,57) = 3.25, p = .08, ηp =.05. As indicated by planned contrasts, backward priming only occurred, when the backward prime succeeded the target by 150 ms (M = 9.4 % vs. M = 11.4 %), F(1,57) = 11.01, p = .002, ηp = .16, but not by 250 ms (M = 8.8 % vs. M = 9.4 %), F(1,57) = 1.11, p = .30.
