# Supplementary material for: Shifting Evaluation Windows: Predictable Forward Primes with Long SOAs Eliminate the Impact of Backward Primes
Source: PLoS One. 2013 Jan 24;8(1):e54739. doi: 10.1371/journal.pone.0054739 (PMC3554650; doi:10.1371/journal.pone.0054739)
Supplement: Supporting Information S2 — Experiment 2 : Replication of Shifting Evaluation Window effect for Blockwise Condition for participants, who received this condition first. (RTF) [file pone.0054739.s002.rtf]

Supporting Information S2:

Table S1. Mean RTs as a function of Forward SOA and Backward Congruency (Experiment 2, Blockwise, for participants, who received Blockwise condition first)
	Backward Prime		
FSOA	Congruent	Incongruent	BEP	
 150 ms	636	647	11†	
250 ms	615	617	2	
Note. BSOA = SOA of Backward Primes; BEP = Backward Evaluative Priming; ** p = .001; * p <.05; †< .10.

Due to the reduction of sample size (i.e. 30 instead of 60, cf. Experiment 1) the crucial Forward SOA x Backward Congruency Interaction itself was not significant; F(1,29) = 0.99, p =.33. However, planned contrasts clearly replicated the expected shifting evaluation window effect: When the forward prime preceded the target at 150 ms, there was a marginally significant backward priming effects, F(1,29) = 4.00, p =.055. In contrast, if the forward prime preceded the target at 250 ms, clearly no backward priming occurred, F = 0.07, p = .80.
