# Supplementary material for: Shifting Evaluation Windows: Predictable Forward Primes with Long SOAs Eliminate the Impact of Backward Primes
Source: PLoS One. 2013 Jan 24;8(1):e54739. doi: 10.1371/journal.pone.0054739 (PMC3554650; doi:10.1371/journal.pone.0054739)
Supplement: Supporting Information S3 — Additional Analysis: The Moderating Role of Congruency between Go Signal Prime and Target [cf. 3] for Experiment 1 and 2. (RTF) [file pone.0054739.s003.rtf]

Supporting Information S3:
The Moderating Role of Congruency between Go Signal Prime and Target [cf. 3]

Experiment 1, Response Times

Table S2. Mean RTs (ms) as a function of Backward SOA, Forward and Backward Congruency (Experiment 1)
		Backward Prime		
BSOA	Forward Prime	Congruent	Incongruent	BEP	
- 150 ms	Congruent	642	667		25**	
	Incongruent	661	668		7	
- 250 ms	Congruent	635	638		3	
	Incongruent	646	648		2	
Note. BSOA = SOA of Backward Primes; BEP = Backward Evaluative Priming; ** p = .001; * p <.05; †< .10. The Backward SOA x Forward Congruency x Backward Congruency Interaction was not significant; F(1,58) = 1.66, p =.20.
Experiment 2, Response Times

Table S3. Mean RTs as a function of Backward SOA, Forward and Backward Congruency (Experiment 2, Blockwise)
		Backward Prime		
BSOA	Forward Prime	Congruent	Incongruent	BEP	
- 150 ms	Congruent	626	646		20*	
	Incongruent	646	643		-3	
- 250 ms	Congruent	622	627		5	
	Incongruent	629	645		16	
Note. BSOA = SOA of Backward Primes; BEP = Backward Evaluative Priming; ** p = .001; * p <.05; †< .10. The Backward SOA x Forward Congruency x Backward Congruency Interaction was significant; F(1,57) = 5.62, p < .05, ηp = .09.


Table S4. Mean RTs as a function of Backward SOA, Forward and Backward Congruency (Experiment 2, Trialwise)
		Backward Prime		
BSOA	Forward Prime	Congruent	Incongruent	BEP	
- 150 ms	Congruent	626	634		8	
	Incongruent	638	639		1	
- 250 ms	Congruent	616	616		0	
	Incongruent	622	631		9	
Note. BSOA = SOA of Backward Primes; BEP = Backward Evaluative Priming; ** p = .001; * p <.05; †< .10. The Backward SOA x Forward Congruency x Backward Congruency Interaction was not significant; F(1,57) = 1.30, p =.26.
